# Synthesis of chemically stable covalent organic frameworks in water

**DOI:** 10.1107/S2052252516016900

**Published:** 2016-10-26

**Authors:** Samantha Yu-ling Chong

**Affiliations:** a Materials Innovation Factory and Department of Chemistry, University of Liverpool, Crown Street, Liverpool, Merseyside L69 7ZD, United Kingdom

**Keywords:** covalent organic frameworks, crystalline porous polymers, dynamic covalent chemistry, microporous materials

## Abstract

The development of environmentally benign and scalable synthetic routes to chemically stable covalent organic frameworks (COFs) is key to their real world application in areas such as gas storage and proton conduction. Banerjee *et al.* [*IUCrJ* (2016), **3**, 402–407] have exploited the high chemical stability of the keto-enamine linkage to develop a ‘green’ water-mediated procedure, presenting a scalable route to chemically robust COFs.

Covalent organic frameworks (COFs) – polymeric materials that form ordered porous architectures – can exhibit a range of compelling properties, such as high gas uptake capacities and facile charge transport, that can be tuned by diversifying the composition of the organic network (Huang *et al.*, 2016 ▸). The prototypical COFs reported by Yaghi and Lavigne (Côté *et al.*, 2005 ▸; Tilford *et al.*, 2006 ▸) were synthesized *via* condensation reactions of boronic acids under solvothermal conditions to form boroxine and boronate ester-linked two-dimensional hexagonal frameworks. The use of reversible bond formation or dynamic covalent chemistry (DCC) is key, as it allows for ‘error correction’ during the assembly of the network. Hence, DCC results in the formation of the thermo­dynamically stable product, promoting an ordered atomistic arrangement in the COF, in contrast to the amorphous nature of most polymers. The structure of the frameworks can generally be characterized by powder diffraction analysis, often in combination with molecular modelling, although the single-crystal structures of a small number of three-dimensional COFs have been determined using X-ray (Beaudoin *et al.*, 2013 ▸) and electron diffraction (Zhang *et al.*, 2013 ▸).

The design and assembly of COFs can be guided by the principles of reticular chemistry (Yaghi *et al.*, 2003 ▸; Côté *et al.*, 2007 ▸; El-Kaderi *et al.*, 2007 ▸; Hunt *et al.*, 2008 ▸), enabling the pore size and shape, and the chemical and electronic properties of the pores and framework to be modulated by selecting appropriate building blocks to form the network. This reticular assembly approach using a variety of DCC reactions, including imination, triazine, hydrazone and azine formation (DeBlase & Dichtel, 2016 ▸; Huang *et al.*, 2016 ▸; Segura *et al.*, 2016 ▸), has led to a proliferation of two- and three-dimensional (El-Kaderi *et al.*, 2007 ▸; Beaudoin *et al.*, 2013 ▸; Uribe-Romo *et al.*, 2009 ▸) COFs. The synthesis of COFs is generally performed under solvothermal conditions in high-boiling solvents, and the use of a sealed system to retain water evolved in the reaction is considered critical to maintaining reversibility during framework formation (Huang *et al.*, 2016 ▸). However, it is also amenable to non-conventional methods, such as microwave-assisted synthesis (Campbell *et al.*, 2009 ▸; Dogru *et al.*, 2013 ▸; Wei *et al.*, 2015 ▸), sonochemistry (Yang *et al.*, 2012 ▸), flow chemistry (Peng, Wong *et al.*, 2016 ▸; Bisbey *et al.*, 2016 ▸) and mechanosynthesis (Biswal *et al.*, 2013 ▸; Das *et al.*, 2014 ▸; Peng, Xu *et al.*, 2016 ▸).

Although COFs can exhibit diverse functional properties that highlight potential applications in areas such as gas capture and storage (Mendoza-Cortés *et al.*, 2010 ▸, 2012 ▸; Zeng *et al.*, 2016 ▸), photovoltaics (Dogru & Bein, 2014 ▸; Wan *et al.*, 2009 ▸), organocatalysis (Xu *et al.*, 2015 ▸) and proton conductors (Chandra *et al.*, 2014 ▸; Shinde *et al.*, 2016 ▸; Xu *et al.*, 2016 ▸), their practical applicability has been hampered by the relativity poor moisture stability, which is related to the reversibility of the covalent linkages used for their formation. Boronate and boroxine linkages, in particular, are prone to cleavage in the presence of water, although imine-linked COFs can exhibit higher hydrolytic and acid/base stability (Xu *et al.*, 2015 ▸; Huang *et al.*, 2016 ▸; Segura *et al.*, 2016 ▸). One approach to further enhance the stability of COFs formed by imination is the incorporation of hydroxyl-functionality in the aldehyde precursor (Kandambeth *et al.*, 2012 ▸) that allows the resultant enol-imine framework to undergo irreversible tautomerism to form the keto-enamine. The reversible imine bond is absent from the keto form of the framework and, therefore, the stability of the COF can be exceptionally high in the presence of water, acid and base (Kandambeth *et al.*, 2012 ▸; Biswal *et al.*, 2013 ▸; Das *et al.*, 2014 ▸).

The recent paper published in **IUCrJ** by Banerjee *et al.* dramatically demonstrates the chemical stability that can be accessed by synthesizing keto-enamine-linked COFs, and exploits this to develop an environmentally benign protocol for the scalable synthesis of COFs in acidified water (Thote *et al.*, 2016 ▸). The synthesis of five previously reported COFs, TpPa-1, TpPa-2, TpBD, TpBpy and DAAQ was reproduced using the hydro­thermal route, and a new framework TpFn (Fig. 1 ▸) containing a fluorene moiety is also reported. Formation of the ordered layered frameworks was indicated by diffraction analysis, which shows significantly improved crystallinity and surface area in comparison to the materials formed by mechanosynthesis – an alternative green synthetic route. The COFs synthesized in water also exhibit the exceptional chemical stability of their mechanochemically and solvothermally synthesized counterparts, and retain Brunaeur–Emmett–Teller surface areas comparable with the solvothermal products. Mechanistic aspects of the keto-enamine formation in water were investigated using time-dependent UV–vis experiments. Using condensation reactions to form analogous COF monomers as a test system, the formation of the imine monomer from tri­aldehyde and aniline precursors was sluggish, which was attributed to the high reversibility of the Schiff base reaction in water. In comparison, reaction of the hy­droxy­lated tri­aldehyde with aniline to form the keto-enamine is relatively facile. The two-step process of reversible imine formation followed by tautomerization to the keto form removes the imine product from the equilibrium, thereby driving formation of the imine and conversion to the final keto-enamine monomer.

The development of water-stable COFs has improved the prospect of their practical use in areas such as proton-exchange membranes (Shinde *et al.*, 2016 ▸) and in CO_2_ capture (Zeng *et al.*, 2016 ▸), for example. In combination with a green, scalable synthetic procedure and diversification of the keto-enamine frameworks, this offers a far broader scope for investigating the potential for more diverse real world applications of COFs, in which moisture and acid/base stability is often a critical factor.

## Figures and Tables

**Figure 1:**
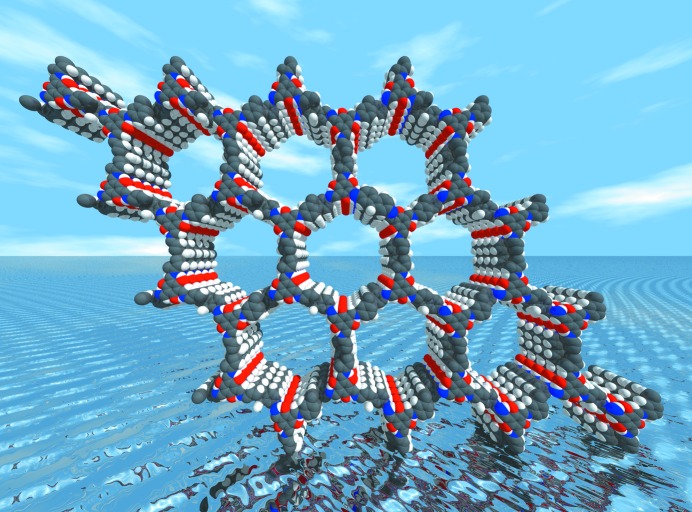
Proposed model of the crystal structure of the keto-enamine-linked covalent organic framework TpFn viewed parallel to [001].
